# Optical fluorescence imaging with shortwave infrared light emitter nanomaterials for in vivo cell tracking in regenerative medicine

**DOI:** 10.1111/jcmm.14670

**Published:** 2019-09-27

**Authors:** Leyla Fath‐Bayati, Mohammad Vasei, Ehsan Sharif‐Paghaleh

**Affiliations:** ^1^ Department of Tissue Engineering & Applied Cell Sciences School of Advanced Technologies in Medicine Tehran University of Medical Sciences (TUMS) Tehran Iran; ^2^ Department of Tissue Engineering School of Medicine Qom University of Medical Sciences Qom Iran; ^3^ Cell‐based Therapies Research Institute Digestive Disease Research Institute (DDRI) Shariati Hospital Tehran University of Medical Sciences (TUMS) Tehran Iran; ^4^ Department of Immunology School of Medicine Tehran University of Medical Sciences Tehran Iran; ^5^ Department of Imaging Chemistry and Biology Faculty of Life Sciences and Medicine School of Biomedical Engineering and Imaging Sciences King's College London London UK

**Keywords:** cell tracking, cell‐based therapy, molecular imaging, optical fluorescence imaging, quantum dots, shortwave infrared, shortwave infrared region

## Abstract

In vivo tracking and monitoring of adoptive cell transfer has a distinct importance in cell‐based therapy. There are many imaging modalities for in vivo monitoring of biodistribution, viability and effectiveness of transferred cells. Some of these procedures are not applicable in the human body because of low sensitivity and high possibility of tissue damages. Shortwave infrared region (SWIR) imaging is a relatively new technique by which deep biological tissues can be potentially visualized with high resolution at cellular level. Indeed, scanning of the electromagnetic spectrum (beyond 1000 nm) of SWIR has a great potential to increase sensitivity and resolution of in vivo imaging for various human tissues. In this review, molecular imaging modalities used for monitoring of biodistribution and fate of administered cells with focusing on the application of non‐invasive optical imaging at shortwave infrared region are discussed in detail.

## 1 INTRODUCTION

Organ failure is a catastrophic phenomenon of many human chronic debilitating diseases. Plasticity and migration capacity of stem cells has opened up new prospects towards treating a wide range of human diseases in recent years and sheds light on expanding fields of regenerative medicine.^1^, ^2^, ^3^, ^4^ Cell‐based therapy is an interdisciplinary field in regenerative medicine, which can treat such disorders by application of therapeutic cells instead of organ transplantation.^5^ The success of cell‐based therapies and their clinical translation to humans depends on two properties of adaptive cell transferred: safety and efficacy.^6^ Despite promising cell therapy studies stating improvement and recovery of damaged organs,^7^, ^8^, ^9^ there are still controversial findings in the literatures regarding effectiveness^10^, ^11^ and safety.^12^, ^13^ Thus, tremendous challenges have been come up in the application of this kind of treatment in regenerative medicine, which are discussed below.

## 2 FACTORS AFFECTING CELL FATE

Biodistribution pattern, viability and fate of therapeutic cells in the target tissue after infusion are main causes of contradictory results among published studies.^14^ Thus, ambiguity in the engraftment site and cell efficacy after transplantation complicates the interpretation of the results from various studies. For cell‐based therapy studies, size of infused cells, routes of cell infusion, cell dosage, infusion rate, time‐point of cell transplantation and host bio‐immunological factors may affect the cell translocation and engraftment to the target tissue.^15^

### 2.1 Cell size

It is suggested that increasing number of cell passages during in vitro expansion leads to the enlargement and widening of the cell size. This issue is considered as one of the important reasons for cell entrapment in lung and obstruction of subsequent small capillaries after intravenous cell infusion.^15^, ^16^, ^17^

### 2.2 Route of cell delivery

Cell delivery route has also a major effect on the localization and fate of transplanted cells in the living body.

#### 2.2.1 Systematic cell delivery

Cell transplantation through the systemic circulation is achieved via intravenous, intra‐arterial and intraperitoneal routes. Various animal studies have demonstrated that the vascular bed of the lung is the first place where intravenously administered cells convene, which can cause small venule obstruction.^14^ Consequently, subsequent interaction with lung vascular endothelial cells affects their viability, biodistribution and clinical efficiency.^16^, ^18^, ^19^, ^20^ Eggenhofer et al studied the viability and biodistribution of intravenously infused mesenchymal stem cells (MSCs) after 5 minutes and 1, 24 and 72 hours. The transplanted cells could be found viable in the lung tissue only in 24 hours, but after 24 hours post‐cell injection, no viable cells in the lung or other tissues such as liver, spleen or heart were found.^21^ Administration of cells through the arterial route can bypass the pulmonary pathway and facilitate the translocation of cells to the intended organs.^15^, ^22^, ^23^ This route of infusion can enhance the cell localization and engraftment at ischaemic brain^24^ and damaged kidneys.^22^ However, intra‐arterial administration of cells may compromise arterial blood supply and cause accumulation in small arteries,^24^, ^25^, ^26^ leading to organ infarction.^24^ Li et al demonstrated that, though, intra‐arterial neural progenitor stem cell delivery produces successful biodistribution and engraftment of infused cells in the brain, but yielded to a significant mortality of animals during the procedure. The reason of high mortality during cell administration may be associated with decreased blood supply to brain parenchyma, predisposing it to ischaemia, thrombosis, oedema, high intracranial pressure and consequently death of animals.^27^ Vulliet et al have investigated the safety of MSC delivery to intracoronary blood flow for treatment of myocardial diseases. They infused MSCs into coronary artery of healthy animal models, and 7 days after cell infusion, healthy dogs exhibited signs of myocardial infarction. Histologic evaluation of myocardial tissue proved acute ischaemia and subacute microinfarction likely due to enlargement of MSC size during in vitro expansion or high dosage of MSCs.^28^ In another study, high percentage of intra‐arterially infused MSCs were entrapped at the precapillary level due to greater size of these cells compared to the diameter of microvessels.^25^ Precapillary occlusion results in blood flow disturbance and ischaemia, which leads to consequent death.^25^ It has been also claimed that MSC infusion through the arterial route can increase the localization of cells to the target tissue (such as ischaemic brain of animal models), but it resulted in failure of functional recovery of the damaged parenchyma.^29^

Surprisingly, low‐dose cell delivery for treatment of ischaemic stroke through intra‐arterial pathway leads to the improvement of inflammation and decreases rate of embolus formation in vessels.^30^

Another undesirable side effect of intra‐arterial cell administration is the fragmentation of infused cells due to the shear forces of arterial blood flow. These damaged cells may be rapidly removed from the circulation through the liver and spleen, causing shorter blood half‐life of infused cells.^15^ Intraperitoneal delivery is another pathway for systematic delivery of cells to the living body with controversial results.^14^ It is thought that cell administration through the intraperitoneal cavity causes circumventing of pulmonary passage and consequently can lead to an increase in the number of transferred cells to the target organs.^31^ However, it has been shown that cell delivery using this route leads to the aggregation of transplanted MSCs with the host immune cells after several minutes. These small and large aggregates adhere on the peritoneal membranes including omentum and mesentery.^32^ These masses cannot enter the blood circulation, and only very small subsets of MSCs that do not aggregate can be visualized in the mesenteric lymph node and spleen in the initial minutes after transplantation. Moreover, no trace of infused MSCs can be found in the other organs such as heart or liver.^32^ Nonetheless, the results of another study emphasize on the localization of transplanted cells in the inflamed colon, which opens up new way for treatment of inflammatory bowel disease using stem cells.^33^

#### 2.2.2 Local injection

Theoretically local infusion of therapeutic cells to the parenchyma may increase the number and retention of the transplanted cells in the target tissue^34^ but with certain concerns.^22^ Local injection in the parenchyma is an invasive method and may lead to further damage to the target tissue.^35^ Direct intramyocardial cell delivery developed cardiac arrhythmias^36^ and had deteriorating effect on the heart.^37^

Conversely, other studies have reported that infusion of high dosage of therapeutic cells directly to the myocardial tissue results in increased localization of transferred cells,^37^ but due to the safety issues related to cell dose, implementation of this technique is not feasible. In addition, direct intraparenchymal cell delivery for treatment of kidney diseases results in accumulation of transplanted cells at the site of infusion and did not distribute throughout the renal parenchyma.^35^ Eventually, administration of large amount of cells into the hepatic parenchyma produced cell embolus formation in the lung.^38^ Surprisingly, there are reports implying that this pathway of injection cannot increase the cell viability and engraftment in target tissues.^22^, ^39^

### 2.3 Time‐point

In addition, time‐point of cell transplantation into damaged tissues can have a significant effect on cellular localization, engraftment and regeneration of damaged tissues.^40^ Erpicum et al demonstrated that timing of administration of MSCs has important effect on outcome of kidney ischaemia/reperfusion (I/R) injury in small animal models.^41^ Findings from their study show that administration of MSCs before I/R injury has nephroprotective effect compared to MSC administration after injury.^41^ MSC infusion before liver damage has significant impact on promoting liver fibrosis. On the contrary, injection of MSCs in resolution phase speeds up liver regeneration.^42^, ^43^

### 2.4 Cell dose

Also, characterization of optimal cell density that can regenerate the damaged tissue without adverse effects such as tumorigenicity^44^ or embolus formation^45^ is controversial and there is no comprehensive consensus on optimal infused cell density.^40^ This lack of consensus is due to several factors that are involved in the determination of cell dose such as type of transplanted cells, recipient's disease and route of cell transplantation.^40^ However, investigators demonstrated that embolic stroke that results from intra‐arterial cell delivery is due to accumulation of cells in the blood vessels and depends on the cell numbers that are transferred.^46^, ^47^

### 2.5 Cell infusion rate

In addition, cell infusion rate must be adjusted in such a timing that maximal cell viability is maintained during injection.^48^, ^49^, ^50^ High injection rates increase shear forces, resulting in cell damages and viability reduction.^15^, ^40^

### 2.6 Host bio‐immunological factors

It is also believed that majority of administered cells may encounter rapid clearance from the body due to the harsh and unfavourable environmental conditions such as anoikis, ischaemia, inflammation^51^, ^52^, ^53^ and host immune reactions.^54^, ^55^ For instance, chronic inflammation at the target tissue may inhibit regeneration process by preventing transplanted cell recruitment to the damaged tissue.^51^ Also, it may lead to the cellular membrane damage through production of free radicals and cytokines.^52^ Consequently, the success and efficacy of cell‐based therapy may be hindered.

**Figure 1:**
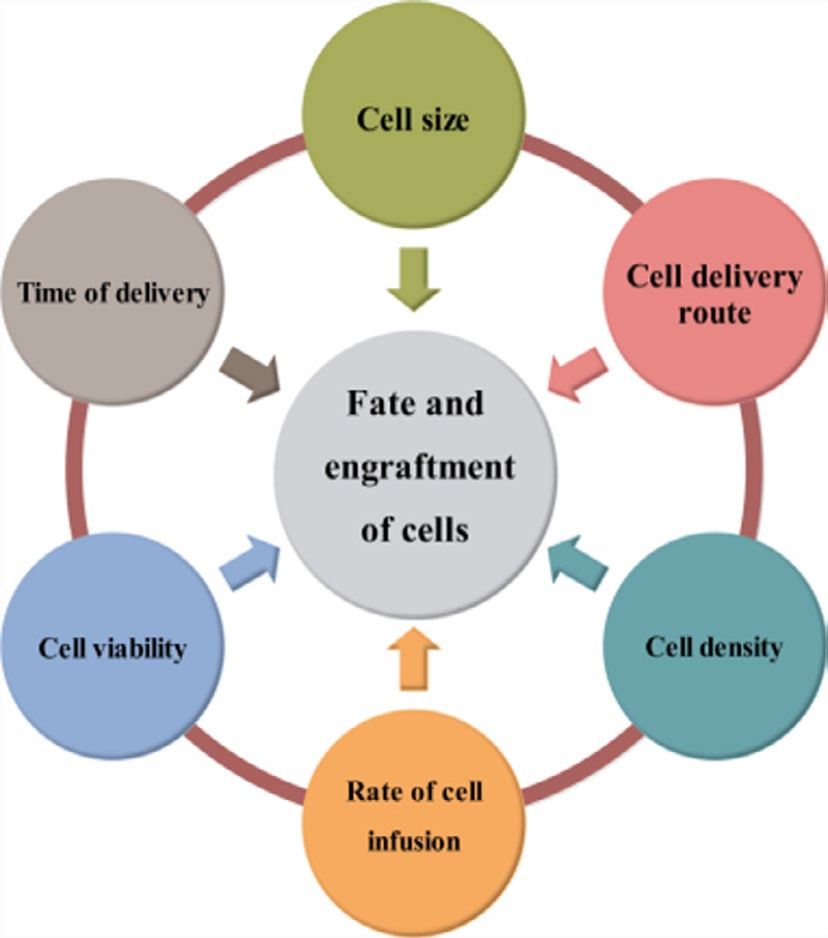
Important factors that affect cell fate and efficacy after administration to living body

In summary, route of migration, biodistribution, dosages, mechanical entrapment of transplanted cells due to enlarged size during successive in vitro expansion, infusion rate and host immunological factors might have detrimental effects on cell engraftment and fate in accordance with Figure 1. Therefore, proper cell tracking and determination of homing by cell imaging is critical to optimize cell administration methods and to characterize the efficacy and safety of cell‐based therapies.^40^

Accurate tracking and in vivo real‐time monitoring of the injected cells will solve the discrepancies between various studies regarding localization, engraftment and interaction of cells with surrounding microenvironment.^56^

## 3 MOLECULAR IMAGING

Information about therapeutic cell function and fate is mostly obtained from fluorescence microscopy and immunohistochemical methods after obtaining biopsy samples from the patients. However, these methods are relatively invasive techniques and may lead to tissue damages and disruption of cellular structures.^57^, ^58^ In addition, these experimental techniques are limited by not being able to trace cells in a real‐time manner.^57^

**Figure 2:**
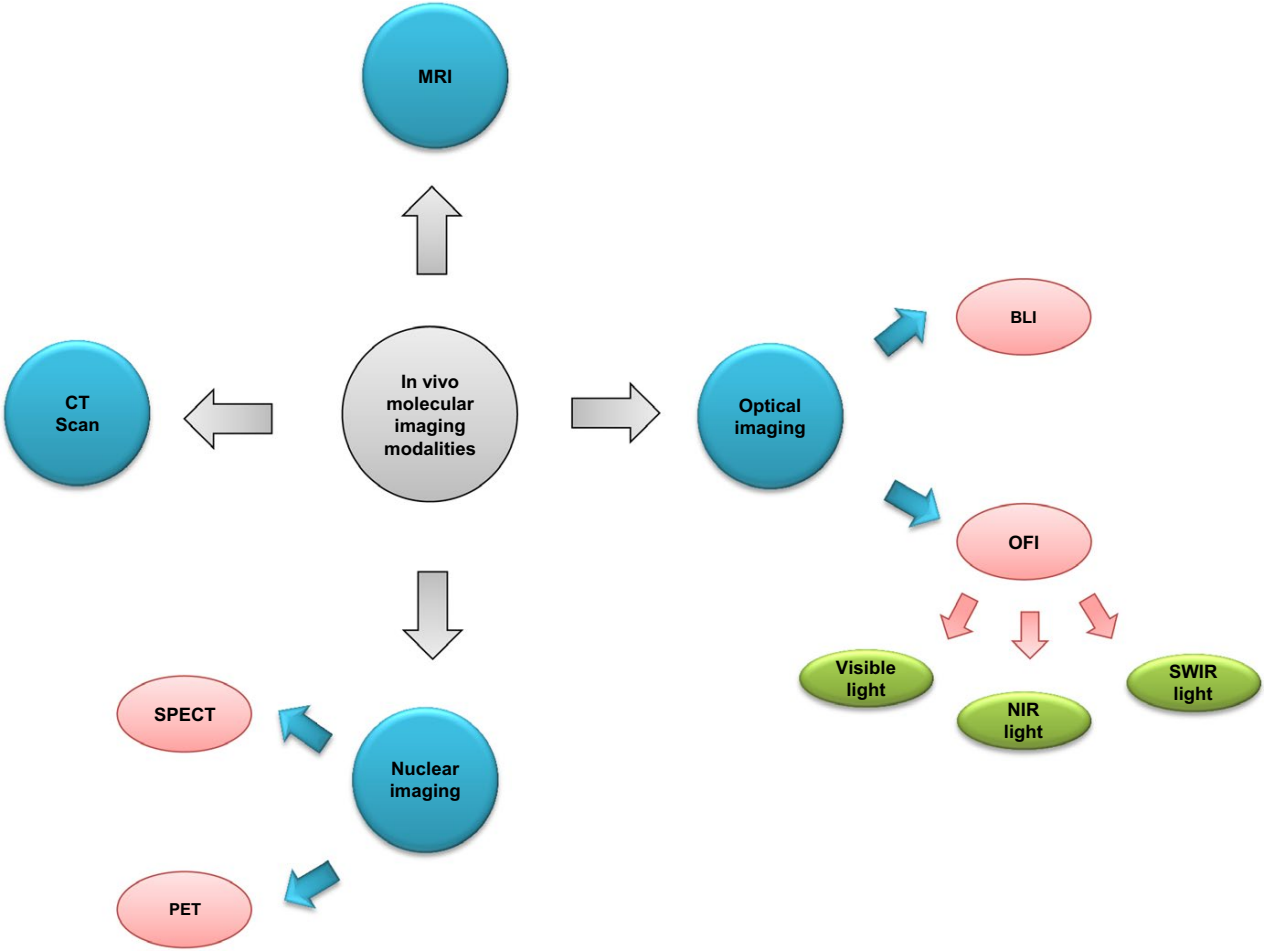
Schematic diagram of in vivo molecular imaging modalities used for cell tracking

Molecular imaging technology is a growing and powerful platform that can provide valuable information about localization site and fate of cells after transplantation.^6^ During the last decades, several in vivo imaging modalities have been developed for researchers to trace delivered cells (Figure 2). However, each of them has its disadvantages that impede their applications as a perfect non‐invasive in vivo imaging technique.^58^ The ideal modality for molecular in vivo imaging must be able to offer accurate information about the survival, biodistribution and engraftment of cells as well as longitudinal functional real‐time response of damaged tissue to cell‐based therapy.^6^, ^59^, ^60^ Furthermore, it also must show a high degree of specificity and sensitivity to obtain information about the adaptively transferred cells without inducing any harmful effects to the body. To address these requirements, it is essential to develop a multifaceted imaging technique that can reach to rapid clinical adoption.^56^

### 3.1 Molecular imaging and cell labelling

To track and monitor translocation and fate of administered cells, target cells have to be labelled by contrast agent or molecular probes that can act as tracers. Two main methods could be used for cell labelling in molecular imaging: direct and indirect labelling. By direct labelling, nanoparticles or chemical agents are delivered into the cell structure prior cell administration into the body. Although the ex vivo labelling of administered cells for various imaging modalities is simple and allows accumulation or internalization of dye in cell surface or internal structure (unless nucleus), there are several challenges. One major obstacle is that intensity of signals produced by labelled cells reduces with cell division over time; thus, direct cell labelling is not appropriate for long‐term tracing of transferred cells in target organs. Other challenges of direct cell labelling are toxicity, bleaching and limited sensitivity of chemical agents used for cell labelling. Indirect labelling is carried out through genetic engineering of cells by reporter genes such as green fluorescent protein (GFP) or bioluminescent luciferase. Genetic modification of cells using exogenous reporter gene that target the cell nucleus results in stable expression of detectable proteins (bioluminescent or fluorescent proteins, enzymes and receptors) in target cells and future progeny. However, this labelling is hampered to find clinical importance due to stable integration of transgene into cellular genome and risk of mutagenesis.^61^, ^62^

#### 3.1.1 Direct cell labelling

Direct cell labelling in molecular in vivo imaging can be done by various compounds including radioactive, paramagnetic or fluorescent agents.^63^ For MRI (magnetic resonance imaging), the nanoparticles consist of superparamagnetic iron oxide (SPIO) nanoparticles, perfluorocarbon nanoparticles, gadolinium‐filled microcapsules and liposomes.^61^, ^64^ Direct cell labelling for nuclear imaging will be implemented with radioisotopes such as ^111^Indium (^111^In)‐oxine or ^99m^ technetium (^99m^Tc) chelates for single‐photon emission computed tomography (SPECT) imaging^65^ and 18F‐fluorodeoxyglucose (FDG) for positron emission tomography (PET).^57^, ^61^For optical fluorescence imaging (OFI), direct cell labelling can be done using lipophilic membrane dyes (including PKH2, PKH26, PKH67, DiD, DiR),^66^ NIR I (near‐infrared region I) and NIR II (near‐infrared region II) emitting fluorophores.^67^ For establishment of various compounds as safe materials for cell labelling, several characteristics are mandatory, including lack of cellular toxicity, optimal renal clearance and stability in biological fluid together with stability during cell division.^62^, ^67^

#### 3.1.2 Indirect cell labelling

The indirect cell labelling allows visualization of the administered cells by the use of various reporter genes such as iron‐storage protein, ferritin, in MRI detection, the herpes simplex virus thymidine kinase type 1 (HSV1‐tk) and human membrane protein sodium‐iodide symporter (NIS) have also been used for positron emission tomography (PET) and hybrid SPECT/CT, respectively.^61^, ^68^, ^69^ For bioluminescent optical imaging, firefly luciferase, *Renilla* luciferase, *Gaussia* luciferase, *Metridia* luciferase, *Vargula* luciferase or *Bacterial* luciferase has been employed as reporter genes.^70^ Finally, indirect cell labelling technique for optical fluorescence imaging is achieved by reporter genes, which express detectable proteins such as green fluorescent protein (GFP).^6^, ^70^

### 3.2 Molecular imaging modalities for in vivo cell tracking

#### 3.2.1 Computed tomography (CT)

Imaging in computed tomography relies on differential absorption of ionizing X‐rays by various tissue components in the body.^71^ However, utilization of the ionizing X‐rays has mutational risks and may damage DNAs.^61^ Necessary instruments for CT imaging include the X‐ray source and rotating detector around the imaged subject.^72^ Low cost compared to other non‐optical imaging modalities and excellent temporal resolution are the advantages of CT scan that make it a potential technique to visualize and track stem cells.^73^, ^74^ The image contrast (differences between attenuation of the X‐ray photons by various tissue) in the CT scan is relatively low for soft tissues; thus, it is imperative to use the contrast agents to distinguish between the various soft tissues.^72^, ^73^ CT scan has potential application in the cell tracking and monitoring particularly in brain and lungs whose development is relatively slower than MRI due to lower contrast of soft tissue.^73^, ^74^

Nonetheless, different studies have shown that gold nanoparticles (AuNP) can be used safely to label, monitor and detect mesenchymal stem cells by conventional CT imaging in vivo.^73^, ^74^, ^75^ However, high dose of ionizing X‐ray radiation requirements is the major disadvantage of CT scan imaging to monitor cellular localization and engraftment.^74^

#### 3.2.2 Nuclear medicine: PET and SPECT

Positron emission tomography (PET) imaging is based on radiotracers that emit positron. After production, radiotracers are unstable, immediately lose their energy and generate some particles named as positrons. These particles interact with neighbouring electrons via annihilation process, and two produced photons (each having 511 keV energy) can be detected by PET scanners.^61^, ^68^, ^76^, ^77^ Cell labelling PET radiotracers include 2‐[F‐18]‐fluoro‐2‐deoxy‐D‐glucose (^18^F‐FDG) and [^64^Cu]‐pyruvaldehyde‐bis (N4‐methylthiosemicarbazone) (^64^Cu‐PTSM). Single‐photon emission computed tomography (SPECT) imaging relies on detection of two low‐energy γ (gamma) photons being emitted from radioisotopes including ^111^In‐oxine and technetium (^99m^Tc) exametazime (^99m^Tc‐hexamethyl propylene amine oxime [HMPAO]).^57^, ^68^

Because penetration in tissue depth in PET and SPECT has no limitation, their cell tracking sensitivity is high, and PET is more sensitive than SPECT.^78^, ^79^

Although labelling procedure of therapeutic cells with PET and SPECT radiotracers is easy in vitro, cell tracking and monitoring should be performed immediately as a result of short half‐life of the agents in vivo. Radiotracers that are currently used in preclinical and clinical studies are removed through liver metabolism and renal clearance.^79^, ^80^, ^81^, ^82^ Despite foregoing advantages of the radiotracers, direct cell labelling has some limitations for in vivo cell monitoring such as disruption of cell viability, impossibility of long time study due to the short half‐life and the leakage of radiotracers into the extracellular area.^57^, ^83^, ^84^ Indirect cell labelling by PET reporter genes, such as herpes simplex virus thymidine kinase type 1 (HSV1‐tk), human nucleoside kinases deoxycytidine kinase (dCK) and thymidine kinase 2 (tk2), compensate the limitations of direct labelling and increase uptake of the radiotracers into cells. However, because HSV1‐tk has non‐human origin its structure induces the immune response in host tissue. In addition, blood‐brain barrier is the main obstacle for intracerebral use of this reporter gene in humans.^57^, ^61^, ^68^ In spite of some problems concerning to genetic manipulations of therapeutic cells, indirect labelling by reporter genes provides a better choice for cell fate tracing in comparison with direct method.^5^ For example, findings from previous study have revealed that NIS reporter gene imaging either by PET or SPECT can be implemented in animal studies for assessment of biodistribution, survival and engraftment of cardiac‐derived stem cells in the myocardium.^78^ But, in spite of high potential of PET reporter gene imaging for cell tracking, application of this technique is restricted to preclinical studies due to low resolution of PET imaging modality at cellular level^85^ and genetic manipulation of transferred cells.^5^

#### 3.2.3 Magnetic resonance imaging (MRI)

Magnetic resonance imaging is a kind of non‐invasive imaging technique that uses a powerful magnetic field to induce polarization of hydrogen nuclei (protons) in water molecules or fluorinated molecules (^19^F). By placing the sample in the magnetic field, the spins polarize towards the main magnetic field. After polarization and alignment of the nucleus, radiofrequency (RF) pulse is applied to the sample that leads to excitation of nuclei and thus causes excitation from lower energy to higher and an unstable state. After removing RF pulse, the nuclei polarize towards the original magnetic field and transit to lower energy state. So, the excess energy of nuclei is released while emitting RF signals being detected by RF coils.^57^, ^58^, ^68^, ^86^ Relaxation period is the duration of the time that takes for the nucleus to transit from high energy level to its basic state. There are three types of the relaxation times in the MRI including longitudinal relaxation time (T1), transverse relaxation time (T2) and T2 without rephasing (T2*).^86^, ^87^ Each tissue component has its own specific relaxation time T1/T2 in the body, which varies between different tissues. The contrast agents in the MRI are classified as T1 and T2 agents, and make differences between various soft tissues. Therefore, in vivo cell tracing and monitoring would be possible for cell‐based therapies by using several MRI contrast media.^68^

Longitudinal relaxation time agents (paramagnetic‐based agents, eg gadolinium) offer positive contrast (bright) by reducing adjacent hydrogen proton T1 relaxation time. T2 agents (iron‐based agents and reporters) reduce the T2 relaxation time of hydrogen protons and offer hypointense (dark) contrast. Other MRI contrast agents include diamagnetic or diamagnetic chemical exchange saturation transfer (DIACEST), paramagnetic CEST (PARACEST) and perfluorocarbon (^19^F) agents.^68^, ^86^, ^88^ Prior to in vivo administration, therapeutic cells must be labelled directly by the MRI contrast agents or indirectly through genetic engineering with the MRI reporter genes such as ferritin, tyrosinase or β‐galactosidase.^5^, ^57^ However, all of these reporter genes used in the MRI cell tracking and visualization do not demonstrate appropriate efficacy.^5^, ^89^ Cell labelling with SPIO and fluorine attracts more attention for future clinical use.^86^ Also, CEST agents could be involved in immunological or other reactions that are not still known.^86^, ^87^ Due to the low sensitivity of PARACEST agents such as Gd^3+^, ensuring the presence of adequate contrast between the various regions in the body requires large amounts of these contrast agents. Thus, application of PARACEST media in higher concentration for long period of time may result in toxic effects. The major limitation related to the cell labelling with the iron oxide nanoparticles is that macrophage engulfs labelled cells after cell death in the body. Thus, approximately 10% of these nanoparticles can be seen in the macrophages and finally lead to misinterpretation of results related to the location and survival of therapeutic administered cells.^5^, ^90^ Indirect labelling of the transplanted cells is implemented by the genetic manipulation via MRI reporter gene, but this method lacks sufficient sensitivity for the cell detection.^68^, ^89^ MRI offers the best anatomical position of the cell graft but lacks adequate information about function, viability and behaviour of the transplanted cells.^90^

#### 3.2.4 Optical imaging

Current in vivo imaging techniques (MRI, PET, SPECT and CT scan) that are used extensively in the clinic for diagnostic purposes are classified as tomographic imaging modalities. They are dependent on deep penetrating radiations such as the X‐ray (CT), high‐energy subatomic particles (PET and SPECT) and strong magnetic fields (MRI).^91^, ^92^ These imaging systems contain some problems such as lack of appropriate spatiotemporal resolution, which is substantial for in vivo single‐cell tracking.^92^, ^93^, ^94^ Optical imaging modalities have been discovered many decades ago for in vitro studies of biological tissues. The extension of these techniques towards non‐invasive in vivo imaging with light photons opens new approaches towards exploring the cellular dynamics and behaviour without harmful effects on the living body.^72^ Optical imaging techniques rely on the detection of transmitted light (photons) through biological tissues.^95^ The light can be generated through two main approaches including bioluminescence (BLI) and fluorescence techniques.^95^

Indeed, therapeutic cells can be labelled indirectly through genetic engineering, using bioluminescent reporter gene such as firefly luciferase or fluorescent reporter gene such as GFP.^96^ Furthermore, cells can be labelled directly by uptaking exogenous fluorophores such as organic dyes and nanoparticles emitting fluorescence light.^62^

##### Optical bioluminescence imaging (BLI)

Optical bioluminescence imaging is based on genetic modification of cells with reporter genes and is considered as a promising method to track cell localization and destiny in live animals. Indeed, bioluminescence imaging relies on the detection of emitted lights from genetically modified cells that express enzyme proteins during chemical reactions in body. Firefly luciferase (isolated from *Photinus pyralis*, North American firefly), *Renilla* luciferase (isolated from *Renilla reniformis*, a click beetle) and *Gaussia* luciferase (isolated from *Gaussia princeps*) are photoprotein‐enzymes that catalyse D‐luciferin substrate in the presence of ATP and O_2_ causing light mission.^5^, ^57^, ^70^, ^97^ By integrating reporter genes with the genome of cells, they can stably express luciferase proteins and can be monitored longitudinally for in vivo imaging. Therefore, bioluminescence imaging does not require additional excitation light source, and light scattering would be minimal due to administration of substrate inside the body. Also, imaging depth of tissue will be possible in live small animals.^5^ Additionally, as mammalian cells do not express endogenous luciferase, in vivo bioluminescence imaging offers the greatest sensitivity compared to tomographic imaging technique.^5^ Furthermore, owing to the generation of bioluminescence signal only in the live cells, biodistribution and fate of live cells can be traced in vivo using BLI. Despite mentioned advantages, the bioluminescence light is attenuated in depth of tissues restricting molecular in vivo imaging to small animal assessment. In addition, immune response and genetic modification are formidable challenges that limit the translation of this technique to clinical studies due to insertion of the reporter gene with the genome of infected cells.^5^, ^57^, ^70^, ^97^

##### Optical fluorescence imaging (OFI)

Labelling of therapeutic cells for optical fluorescence imaging can be done through indirect or direct labelling.

Optical fluorescence imaging using indirect labelling, as mentioned above, was implemented by genetic engineering of target cells to express fluorescent reporter proteins. Fluorescent reporter gene techniques rely on fluorochromes such as green fluorescent protein (GFP) that is excreted from Aequorea Victoria jelly fish as a by‐product uncovered in 1961. Other fluorescent proteins include fluorochromes, which emit red and far‐red light and also mutant forms of the GFP gene that emit yellow or cyanin light.^57^, ^72^, ^98^, ^99^ Attenuation of excitation and emission wavelengths of fluorescent reporter proteins such as GFP, due to scattering and absorption by the biological tissue, impedes further penetration of photons. Consequently, signal generation becomes weak and this technique cannot be implemented for in vivo tracking and monitoring of administered cells in live animal models. Other obstacles such as immunogenicity and toxicity of GFP along with weak signal and genetic manipulation of cells cause a limited application of this protein to ex vivo analysis of therapeutic cells and post‐mortem immune histochemical evaluation of excised tissues.^57^, ^72^, ^98^

Direct labelling of therapeutic cells for OFI generally consists of in vitro cell labelling prior to in vivo administration by fluorophores (fluorescent probe) or nanoparticles. Then, labelled cells are excited using photons in defined wavelengths of spectral region and emitted light from cells is detected with high sensitive detector or camera.^97^ Aside from limited length of penetration in deep biological tissues,^5^ beneficial effects of fluorophores in vivo include cost‐effectiveness, high sensitivity and high spatial resolution necessary for cell monitoring.^100^ OFI is one of the most promising modalities that can open new ways for non‐invasive in vivo cell visualization without the use of ionizing radiation.^62^ However, this modality is hampered by the light scattering and absorption along with inherent tissue autofluorescence that corrupt signal detection by detector due to the high ratio of background noise to signal in the visible region (between 400 and 650 nm) of electromagnetic spectrum. Autofluorescence of a tissue mostly emanates from NADPH, flavins and collagen.^101^ Translocation of light photons through a turbid media, such as biological tissues that consisting of endogenous chromophores, eventuates in three main components: diffusive, ballistic and snake photons. The ballistic and snake photons consist of beneficial information, but the diffusive photons make some noise and lack useful data for imaging procedure due to the haphazard scattering.^102^, ^103^

Major endogenous chromophores (light absorbers) that significantly absorb the light in the visible light include water, lipids, oxyhaemoglobin and deoxyhaemoglobin that particularly has a high absorption peak in the visible region of the spectrum.^100^, ^102^, ^104^, ^105^, ^106^ Also, the morphology, size and composition of tissues can act as light scatter.^104^ The scattering and absorption features of the light in the turbid media, for example human body, result in disruption of image contrast. Reduction of image contrast with increasing the tissue depth depends on the issues including blurring of images and reduction of photons. Scattering of photons that haphazardly transmit through biological tissue makes images blurry, and absorption by different components of tissue reduces detectable photons.^104^ Estimation of the scattering and absorption can be performed by energy Beer‐Lambert's intensity law.^102^ Therefore, increasing the depth of tissue in the living body negatively affects the contrast and finally leads to the reduction of sensitivity and spatiotemporal resolution of the image.^102^ So, one of the main goals of in vivo optical imaging is increasing the depth of photon penetration in biological tissues.

## 4 BIOIMAGING IN SWIR REGION

The use of visible region of electromagnetic spectrum in the range of 400‐650 nm is suitable to get image from accessible or superficial tissues such as colon and skin, but not for structures locating in the deeper parts of the body such as nucleus or stem of the brain due to the scattering and absorption by tissue components.^107^

During the last two decades, many efforts have been made to increase image contrast by diminishing between the tissue scattering and absorption of light photons along with reducing disruptive autofluorescence signals due to increasing the tissue depth to avoid potential deleterious effects of tissue parameter on in vivo fluorescence imaging.^108^

The results of the various studies demonstrated that longer wavelength lights have more penetration depths than the visible light. This phenomenon is due to the decrease in photon scattering and absorption by biological tissues. Extending optical fluorescence imaging from the visible region (400‐650 nm) to near‐infrared region of the spectrum (650‐900 nm, called NIR I optical window or therapeutic window) offers considerable improvement in the image contrast compared to fluorescent imaging in the visible region. Indeed, by using longer wavelengths in the NIR I window, the transparency of opaque tissue will increase as a result of the better penetration of photons to the tissue media. Also, at the longer wavelengths, autofluorescence of the biological tissue does not visualize or is negligible.^109^, ^110^, ^111^ By using the NIR I biological window for imaging purposes, non‐invasive in vivo fluorescence imaging of different organs and monitoring of cell‐based therapy are possible.^112^ Furthermore, it is beneficial for medical utilization such as optical spectroscopy due to the use of longer wavelengths of spectra along with accessible and cost‐effectiveness of silicon‐based detectors.^113^

**Figure 3:**
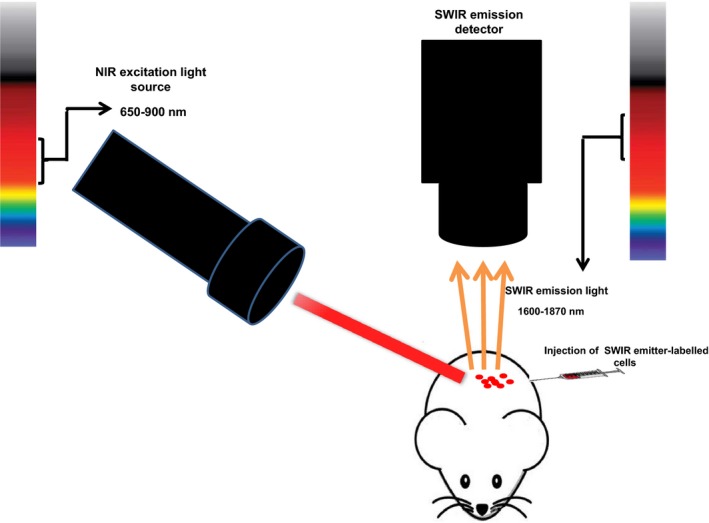
Schematic illustration of optical instrumentations for cell tracking with SWIR light

Optical spectroscopy using exogenous fluorophores that emit light in the range of NIR I window is extensively used in the clinic as an important diagnostic method to evaluate blood flow inside the brain and determine tumour margin for precise resection and removal of cancerous tissue during surgery.^114^, ^115^ In addition, imaging by optical properties of tissue and by endogenous tissue chromophores such as lipid, water and collagen contents can be a valuable method in label‐free studies. It can help to diagnose malignant overgrowth from benign or normal tissue structure.^114^, ^115^, ^116^, ^117^, ^118^ Also, technical advances such as sensitive detectors in NIR region, laser light sources and life science technologies can eliminate mutational risk percentage in optical mammography.^114^, ^115^ In addition, NIR I fluorophores possess applicable quantum yield and high resistance against photobleaching and chemical degradation. So, these agents can be utilized to label various kinds of cells to visualize cellular dynamics and fate in the living body.^119^ Thus, optical imaging in NIR I region can play a crucial role in tailoring infused cells by assessing their localization and viability. However, the major limitation of optical fluorescence imaging for clinical translation is still the limited depth of light penetration and poor spatial resolution due to the high scattering.^62^, ^120^ Despite possibility of non‐invasive in vivo NIR I optical imaging, obtaining clearer image with increasing depth of tissue cannot be optimal choice due to the high level of light scattering by biological tissues.^121^ Light scattering in the tissue depth eventuates higher background noise‐to‐signal ratio and minimizes the sensitivity of NIR I light to deep scanning of tissue.^121^ Thus, for further penetration of light inside the opaque tissues, imaging in the NIR I optical window cannot satisfy the clinical needs. Acquired data from water absorption characteristic in NIR I region and longer wavelength regions show a strong peak in these regions that consequently leads to reducing in image contrast.^115^, ^118^ Image contrast depends on the absorption and scattering of light photons, and in the visible and NIR I region of electromagnetic spectrum, scattering phenomenon in tissue is a Mie‐type.^115^, ^118^, ^121^ The previous studies have demonstrated that the scattering phenomena can be decreased by using longer wavelengths beyond 1000 nm.^115^, ^121^ Hence, NIR I optical imaging extends to longer wavelengths known as short wave infrared (SWIR) region results in better penetration of light to the opaque tissues. It is good to be noted that wavelength of SWIR biological window is approximately between 1000 and 2500 nm.^122^ In addition to the decrease in the scattering of light in the SWIR region, autofluorescence emanated from biological tissue has reached to minimal level or could not be seen.^104^, ^106^ There are three other biological windows in the SWIR region: NIR II window (second window, at 1100‐1350 nm), NIR III window (third window also called golden window, at 1600‐1870 nm, ideal for brain imaging) and NIR IV window (fourth NIR window, ranging from 2100 to 2350 nm, suitable for the optical imaging of bone).^122^ The third window is called as the golden window because the transparency of brain tissue is maximum in this region of the spectrum due to the higher absorption of lipid in comparison with other windows.^113^, ^122^ In extended NIR (SWIR region), the length of the light photon penetration in depth is fundamentally greater than NIR I window. This is demonstrated by applying exogenous fluorophores such as single‐wall carbon nanotube (SWCNT) at beyond 1000‐nm wavelengths.^118^, ^123^, ^124^ Zhang et al determined the depth of light penetration in opaque tissues from the SWIR region at wavelengths of 900 nm to 1650 nm by using hyperspectral imaging in combination with estimation of spatial Michelson contrast. Their results demonstrated that biological imaging in the SWIR region by wavelengths of 1300 to 1375 nm offers the optimal depth of photon penetration and consequently greater transparency of turbid biological media. In spite of these measurements, they were not able to determine the contrast in longer wavelengths beyond 1650 nm due to the lack of highly sensitive camera.^107^ In another study by Sordillo et al, total attenuation length of different tissues in the SWIR windows showed higher lengths of tissue transmittance of SWIR light in the sample, in comparison with NIR I light. Results of their study showed that as the lipid is the major chromophore in the second and third NIR windows, these regions can be optimal for the imaging and studying organs containing lipids such as brain, normal prostate and normal breast. Also, third and fourth windows are appropriate for normal and abnormal bone tissue assessments because of higher collagen content of bone, which acts as the main chromophore and has large absorption peak.^122^ It has been demonstrated that deep tissue imaging could be possible using SWIR optical imaging due to deep photon penetration that allows higher resolution imaging compared to other modalities.^122^ Non‐invasive in vivo optical imaging in the SWIR region is in its beginnings and should be explored by further efforts. Deeper penetration of photon is necessary for appropriate spatial and temporal resolution at the cellular level that is an essential prerequisite for more advances in the cell‐based therapies. Further advances in optical imaging using the SWIR region of spectra rely on development of powerful laser sources, sensitive camera and suitable SWIR emitter fluorophores.^68^, ^92^, ^107^, ^118^, ^122^, ^125^ Until 2014, development of in vivo optical imaging in SWIR region had been prevented mainly due to the lack of high sensitive, low‐cost, high quantum yield detectors (cameras) and SWIR emitter fluorophores together with advanced laser source. Thus, SWIR technology encountered with several issues that led to the restricted development of this field. This may be mainly due to regulations pertaining to national defence such as International Traffic in Arms Regulations (ITAR).^126^ Recently, by eliminating borders in the application of high sensitive indium gallium arsenide (InGaAs)–based detectors in research, along with super‐continuum laser technology, imaging via SWIR opens new prospects to accelerate the applications of this technique for non‐invasive in vivo tracing of administered cells **(**Figure 3).^125^, ^126^ As various kinds of therapeutic cells do not have sufficient fluorescence particularly in SWIR range, these cells should be labelled with SWIR emitter materials to be distinguished from surrounding area (Figure 3). Same as NIR I fluorophores such as indocyanine green (ICG) and methylene blue (MB) that are used in clinic, several factors are prerequisite for SWIR fluorophores to be approved by Food and Drug Administration (FDA) for preclinical and clinical studies. These factors include suitable renal clearance, lack of any cellular toxicity or photobleaching, stability in biological fluids and emitting in SWIR windows of spectrum with high quantum yields that make optimal fluorescence imaging available.^68^, ^92^, ^125^, ^127^ However, extensive implementation of in vivo SWIR optical imaging for clinical use has been impeded by a lack of bright, non‐toxic fluorophores with high quantum yield.^128^ Various studies^129^, ^130^, ^131^, ^132^, ^133^, ^134^, ^135^, ^136^, ^137^, ^138^, ^139^, ^140^, ^141^ that aimed to develop SWIR fluorophores involved production of several compounds including inorganic carbon nanotubes,^129^, ^130^, ^131^, ^132^, ^133^, ^134^, ^135^, ^136^ various types of quantum dots (Ag_2_S, Ag_2_Se, InSb and InAs‐based quantum dots),^137^, ^138^, ^139^, ^140^ rare‐earth nanoparticles,^104^ IR‐polyethylene glycol (PEG) nanoparticles,^141^ organic CH1055‐PEG molecule^91^ and Pt nanowires.^142^, ^143^

In spite of various SWIR emitting fluorophore production during recent years, quantum yields of these fluorescent probes are very low.^144^ Therefore, construction of SWIR emitting materials that offer better efficiency in the SWIR region is highly needed. For example, almost all of the fluorophores that are utilized for optical imaging in NIR II biological window have a very low quantum efficiency and cannot produce longer wavelength light photons. This leads to shallow penetration of light photons into the biological tissues^145^ and limits SWIR imaging to small animal models.

An interesting published study has revealed the beneficial effects of narrow‐range quantum dots (QDs) for fluorescence in vivo imaging purposes.^125^ Results of this work demonstrated that application of QDs that emit light photons in SWIR region of spectra is a promising approach for deep tissue imaging in preclinical and intravital microscopy (IVM) studies. Nonetheless, for quantum dots to be useful fluorophores for the preclinical applications including cell labelling, a complete study of their biocompatibility and long‐term optical efficiency is necessary because of the presence of toxic heavy metals in chemical composition of QDs.^125^ Thus, the rational design of quantum dots or other molecular probes that lack any toxicity along with high quantum yield is critical for obtaining clear images.^125^, ^128^

### 4.1 Tracking of cell fate using SWIR light

Optical fluorescence imaging is expected to contribute to the development of cell‐based therapy because it can detect labelled cells with high resolution.^6^, ^146^, ^147^ However, tracing of the cell translocation and fate in vivo using conventional fluorescent dyes and reporter genes as mentioned earlier is almost impossible. This is due to the limitations mainly emanated from the inherent autofluorescence characteristic of biological tissues, scattering and absorbance of light passing through the living body.^73^, ^148^ In the last years, several types of nanoparticles, mainly QDs, represent satisfying properties such as suitable resolution and sensitivity and adjustable emission in the SWIR (NIR II) regions for in vivo monitoring of administered cells. Then, they can be used as good replacements for conventional fluorescent agent that emit light in the visible or NIR I regions.^94^, ^149^ For example, Chen and coworkers evaluated tropism of mesenchymal stem cells for cutaneous wound healing in the small animal model using Ag_2_S quantum dots that emit light in the biological SWIR window. They demonstrated the dynamic process of Ag_2_S QD‐labelled MSC biodistribution and homing in response to SDF‐1α on the cutaneous wound healing.^94^ Considering high sensitivity and resolution at the cellular level for optical imaging in the SWIR region, researchers also can evaluate the tumour cell deposits in its early stages in addition to monitoring of the dynamic cellular behaviour.^150^, ^151^ For this reason, Tao et al have investigated the growth of the tumour in the early stage in the small animal model. They initially implanted the ovarian cancer cells in the intraperitoneal cavity of the animal model. After two weeks, they visualized the early tumour deposits using nanoparticles that emit signals in the NIR II region. The tumour deposits were undetectable by using exogenous NIR I fluorophores or intrinsic LUC and red fluorescent protein (RFP) reporter genes that conventionally used to monitor tumour growth and tumour response to various therapies. The promising results of this study can provide an innovative method to image various tumour cells in the early stages of growth by SWIR imaging technology.^150^ Moreover, it is promising that using further achievements in the field of SWIR imaging, investigators can detect the cancer cells that metastasize to the other surrounding tissues. Also, according to the results of this study, it is promising that researchers can accurately assess the safety and potential tumorigenicity of the infused cells after cell administration.

Despite several promising studies in the SWIR region of spectrum related to therapeutic cell visualization and detection, higher efficient and biocompatible SWIR emitting fluorophores are needed for further advancement in this field of molecular imaging. Consequently, SWIR‐based imaging can open up new ways towards non‐invasive in vivo fluorescent imaging with high spatial and temporal resolution at cellular levels.

## 5 CONCLUSIONS

Because there are critical challenges related to the translation of cell‐based therapy to the clinic, in vivo tracking of infused cells for the purpose of cell engraftment and fate is essential for obtaining insights about therapeutic cell efficacy. With this respect, optical fluorescence imaging in SWIR windows (extended NIR) is thought to be a great imaging modality due to its inherent increased lengths of light penetration in non‐homogeneous and opaque tissues. Thus, researchers can achieve better image contrast and high spatiotemporal resolution necessary for in vivo cell tracking using this modality. There has been focused on applications of SWIR‐based in vivo imaging during the last years due to lack of destructive ionizing radiation compared to other imaging modalities (such as PET, SPECT and CT) along with other mentioned advantages (Table 1). Therefore, optical imaging in SWIR region of the electromagnetic spectrum is currently being pursued as a potential replacement for conventional imaging technique. However, our prospect of cell tracing using SWIR imaging is that this modality can be critical in addressing of obstacles related to acceleration of cell‐based therapy to clinic. Further evolution in the SWIR emitter fluorophores could allow researchers to obtain high‐quality images that lack artefacts at cellular level from tissue depth without causing harmful effects on living body.

**Table 1:** Different imaging modalities for monitoring of administered cells

Imaging technique	Spatial resolution (voxel size)	Penetration depth	Advantage	Disadvantage	References
CT Scan	<1 mm^3^	40 cm	Deep penetration 3D imaging, high resolution	Ionizing radiation, low sensitivity	62, 65, 152, 153
PET	~3‐5 mm	50 cm	Deep penetration, high sensitivity, Assessment of cell viability, 3D imaging,	Ionizing radiation, long acquisition time, low spatial resolution	6, 61, 152, 153, 154
SPECT	~5 mm^3^	50 cm	Deep penetration, 3D imaging	Ionizing radiation, long acquisition time, low spatial resolution, ^111^In causes damage to labelled cells	6, 61, 152, 155
MRI	~<1‐3 mm^3^	50 cm	Deep penetration, 3D imaging, absence of haphazard radiation	False‐positive results, low sensitivity, long acquisition time	5, 6, 61, 152, 153
Optical bioluminescence imaging	5‐20 mm	1‐2 cm	No ionizing radiation, relatively high spatial resolution, high sensitivity, assessment of cell viability, absence of background noise	Low penetration depth, low resolution, genetic manipulation	5, 62, 65, 154
Optical fluorescence imaging at visible region	2‐20 mm	~1 cm	No ionizing radiation, relatively high spatial resolution, high sensitivity	Low penetration depth, low resolution	62, 65
Optical fluorescence imaging at SWIR region of spectrum	~25 µm	up to ~3 cm	Lower light scattering or absorption, negligible autofluorescence, higher signal‐to‐noise ratio and consequently higher image quality compared to visible and NIR I region	Low penetration depth, lack of FDA‐approved fluorophores for clinical use	125, 149, 156

## CONFLICT OF INTEREST

The authors declare that they have no conflicts of interest.

## AUTHOR CONTRIBUTIONS

LF contributed to the literature research, drafting, interpretation and writing of manuscript. MV and ES contributed to the supervision, drafting, interpretation and writing of manuscript. ES gave the final approval of the article to be published. Also, all authors critically revised the manuscript draft.
